# Deconvolution of a Large Cohort of Placental Microarray Data Reveals Clinically Distinct Subtypes of Preeclampsia

**DOI:** 10.3389/fbioe.2022.917086

**Published:** 2022-07-13

**Authors:** Tian Yao, Qiming Liu, Weidong Tian

**Affiliations:** ^1^ State Key Laboratory of Genetic Engineering and Collaborative Innovation Center for Genetics and Development, Department of Computational Biology, School of Life Sciences, Fudan University, Shanghai, China; ^2^ Human Phenome Institute, Fudan University, Shanghai, China; ^3^ Children’s Hospital of Fudan University, Shanghai, China; ^4^ Qilu Children’s Hospital of Shandong University, Jinan, China

**Keywords:** deconvolution, preeclampsia, heterogeneity, single-cell, pipeline

## Abstract

It has been well established that the dysfunctional placenta plays an important role in the pathogenesis of preeclampsia (PE), a hypertensive disorder in pregnancy. However, it is not well understood how individual cell types in the placenta are involved in placenta dysfunction because of limited single-cell studies of placenta with PE. Given that a high-resolution single-cell atlas in the placenta is now available, deconvolution of publicly available bulk PE transcriptome data may provide us with the opportunity to investigate the contribution of individual placental cell types to PE. Recent benchmark studies on deconvolution have provided suggestions on the strategy of marker gene selection and the choice of methodologies. In this study, we experimented with these suggestions by using real bulk data with known cell-type proportions and established a deconvolution pipeline using CIBERSORT. Applying the deconvolution pipeline to a large cohort of PE placental microarray data, we found that the proportions of trophoblast cells in the placenta were significantly different between PE and normal controls. We then predicted cell-type-level expression profiles for each sample using CIBERSORTx and found that the activities of several canonical PE-related pathways were significantly altered in specific subtypes of trophoblasts in PE. Finally, we constructed an integrated expression profile for each PE sample by combining the predicted cell-type-level expression profiles of several clinically relevant placental cell types and identified four clusters likely representing four PE subtypes with clinically distinct features. As such, our study showed that deconvolution of a large cohort of placental microarray provided new insights about the molecular mechanism of PE that would not be obtained by analyzing bulk expression profiles.

## 1 Introduction

Preeclampsia (PE) is a hypertensive disorder of pregnancy and is the main reason for maternal and fetal morbidity and mortality (Bokslag et al., 2016). Abnormal development and dysfunction of the placenta are thought to be the main cause of PE though detailed pathophysiology is still not fully understood (Horii et al., 2021). As the placenta is a heterogeneous tissue consisting of diverse types of cells, single-cell studies of PE’s placentas are expected to lead to a better understanding of the molecular mechanisms underlining PE pathogenesis. However, most PE transcriptome studies published so far were done at the bulk level (Leavey et al., 2016; Robineau-Charette et al., 2020; Yadama et al., 2020; Xu et al., 2021). A recently published single-cell study on the placenta of PE included only three samples each in the PE and the control groups (Zhang et al., 2021), providing a limited number of samples to investigate the association of individual cell types in the placenta with PE. Cell-type deconvolution is a technology that can infer cell-type proportions from bulk transcription profiles when cell-type-specific expression profiles of marker genes are available (Jin and Liu, 2021). Given that the high-resolution single-cell atlas of the placenta is now available (Suryawanshi et al., 2018; Vento-Tormo et al., 2018), reanalyzing existing bulk PE transcriptome data by deconvolution may therefore provide us with the opportunity to investigate the contribution of individual placental cell types in the placenta to PE.

Numerous deconvolution methods have been developed (Newman et al., 2015; Hao et al., 2019; Newman et al., 2019; Tsoucas et al., 2019; Wang et al., 2019; Dong et al., 2021), and they can be generally divided into two broad categories (Cobos et al., 2020): the bulk and the single-cell reference-based methods, respectively, with the former requiring a predefined cell-type-specific signature gene matrix and the latter not. CIBERSORT (Newman et al., 2015) and CIBERSORTx (Newman et al., 2019) are the representative methods of these two categories, respectively. The use of deconvolution methods has greatly accelerated the study of diseases. For example, prognostic biomarkers of renal cell carcinoma were identified by estimating the proportions of tumor-infiltrating immune cells by cell-type deconvolution using CIBERSORT (Zhang et al., 2019). Recent benchmark studies evaluating the performance of current deconvolution methods (Cobos et al., 2020; Jin and Liu, 2021; Nadel et al., 2021) have provided suggestions on the strategy of marker gene selection and the choice of deconvolution methodologies. For our study, i.e., conducting deconvolution on bulk PE transcriptome data, however, on the one hand, the detailed thresholds for marker selection need to be specified. On the other hand, we still need to decide on one of several recommended methods to perform deconvolution.

In this study, we followed the strategy suggested by Francisco et al. (Cobos et al., 2020) to determine the thresholds for marker gene selection. Then, by using different sources (RNA-seq and microarray) of real bulk data with known cell-type proportions, we evaluated several deconvolution methods recommended by Francisco et al. using two measures—the Pearson correlation coefficient between the predicted and true cell-type proportions (PCC_P_) and the Pearson correlation coefficient between the predicted and true bulk transcripts (PCC_T_). As PCC_T_ can be directly calculated from a deconvolution, it has been suggested to be potentially useful for improving the performance of deconvolution (Newman et al., 2015; Dong et al., 2021). We, therefore, investigated the relationships between the two PCCs to explore the possibility of using PCC_T_ to select a deconvolution method. Finally, we applied the deconvolution pipeline derived from the above-described experiments to a large cohort of PE microarray data that have detailed clinical phenotypes (Leavey et al., 2016). We then conducted an in-depth analysis on the deconvolution results and particularly explored the cell-type-level expression profiles predicted based on the estimated placental cell-type proportions. Our results led to four PE subtypes with clinically distinct features that would not be observed by analyzing bulk gene expression profiles.

## 2 Results

### 2.1 The Development of a Practical Pipeline for the Deconvolution of Placenta Microarray Data

The benchmark study by Francisco et al. (Cobos et al., 2020) provided suggestions on marker gene selection and the choices of methodologies. For marker gene selection, it is recommended to use a stringent selection strategy by using the following three measures—logFC, logCPM, and SecondFC, representing the cell-type-specificity across all cell types, the averaged expression level across all cell types, and the cell-type to cell-type difference of a marker gene, respectively (see Section 3 for details about the definition of these three measures). For the choice of methodologies, it recommended several bulk reference-based methods, including CIBERSORT (Newman et al., 2015), robust linear regression (RLR) (Venables and Ripley, 2002), FARDEEP (Hao et al., 2019), OLS (Chambers et al., 1990), and nonnegative least squares (NNLS) (Katharine et al., 2012), and several single-cell reference-based methods, including DWLS (Tsoucas et al., 2019), MuSiC (Wang et al., 2019), and SCDC (Dong et al., 2021). We added CIBERSORTx (Newman et al., 2019), which is based on CIBERSORT’s improved method of using single-cell data as input. There are also nonreference-based deconvolution methods available, such as ssFrobenius (Gaujoux and Seoighe, 2012). However, Avila Cobos et al. (2018) had shown that reference-based methods would work better than nonreference-based methods when the reference expression profiles are available. Because the single-cell reference of the placental atlas is available in this study, we did not consider the nonreference-based deconvolution methods in this study. Although the above suggestions were useful, in our case, we still need to determine the thresholds for the three marker gene selection measures and also have to choose a method from the recommended ones.

**FIGURE 1:**
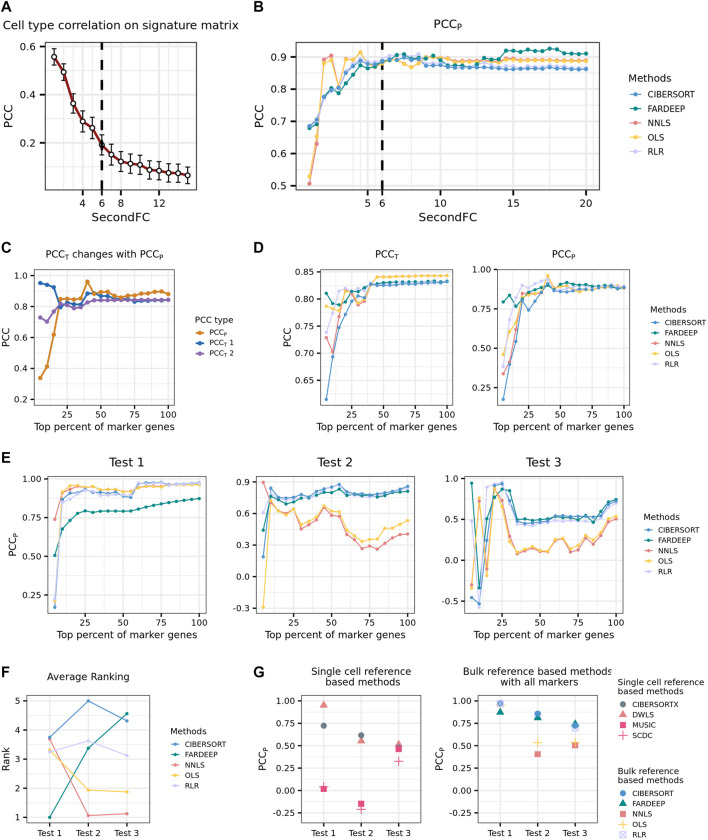
Development of a practical pipeline for the deconvolution of placenta microarray data. **(A)** Average PCC on PBMC signature matrix changing with SecondFC cutoff. **(B)** PCC_p_ of different methods changing with SecondFC cutoff. **(C)** The changes of PCC_T_ and PCC_p_, where the predicted expression profiles of the former and the latter were computed by using the input signature gene matrix varied. PCC_T1_ and PCC_T2_ are the PCC between the predicted and the real bulk expression profiles on inputted signature gene matrix and the signature gene matrix with all marker genes, respectively. **(D)** The changes of PCC_T_ and PCC_p_ by using different deconvolution methods, where PCC_T_ refers to PCC_T2_ in **(C)**. **(E)** Three benchmark tests to evaluate the performance of different deconvolution methods. In Tests 1 and 2, the reference expression profiles were from the 10X scRNA-seq PBMC data generated by Ding et al. (2020), and the bulk data were Finotello's PBMC RNA-seq data and Newman's PBMC microarray data. In Test 3, the bulk data were the same as in Test 2, while the reference expression profiles were the Drop-seq and inDrops scRNA-seq PBMC data generated by Ding et al. (2020) **(F)** The average rank of different deconvolution methods across the three tests in **(E)**. **(G)** The comparison of the performance of single-cell and bulk reference-based methods across the three tests in **(E)**.

To determine the thresholds for marker gene selection, we selected the peripheral blood mononuclear cells (PBMCs) bulk data produced by Finotello et al. (2019) in which cell-type proportions were determined by flow cytometry for deconvolution. We then obtained the reference expression profiles of the immune cell types from the RNA-seq data generated by Hoek et al. (2015) to generate the signature gene matrix. We fixed the thresholds of both logFC and log CPM to be one and experimented with different thresholds of SecondFC to construct the signature gene matrices. We used the Pearson correlation coefficient between the predicted and true cell-type proportions (PCC_P_) for evaluating the performance of deconvolution. We found that with the increase of SecondFC, the average correlation between cell types in the signature gene matrix decreases, but PCC_P_ increases; when the similarity decreases to an inflection point, PCC_P_ would reach a high level (Figures 1A,B). Accordingly, the threshold of SecondFC could be determined by investigating the relationship between SecondFC and the average correlation between cell types in the signature gene matrix.

Next, we aimed to determine which deconvolution method should be used in practice. Given the estimated cell-type proportions by a deconvolution method, the predicted expression profiles of bulk transcripts can be computed by T=C· P , where T represents the predicted bulk expression profile, C is the signature gene matrix, and P is the estimated cell-type proportions. The PCC between the predicted and true expression of bulk transcripts (PCC_T_) can then be calculated. It is assumed that the closer the estimated cell-type proportions to true cell-type proportions, that is, a higher PCC_P_, the closer the predicted expression of bulk transcripts to true expression, that is, a higher PCC_T_. It has thus been proposed that maximizing PCC_T_ may have the effect of maximizing PCC_P_ (Newman et al., 2015; Dong et al., 2021). If this were true, then PCC_T_ may also be used for selecting the deconvolution method, that is, a method with a greater PCC_T_ ought to have a greater PCC_P_. To test this possibility, here we investigated the relationships between PCC_p_ and PCC_T_.

From the marker genes selected by following the above-described parameters, we selected a top fraction of genes according to their logFC to generate a signature gene matrix and conduct deconvolution. A pair of PCC_p_ and PCC_T_ could be calculated for each selected fraction of marker genes, and a series of paired PCC_p_ and PCC_T_ could be calculated by increasing the fraction of marker genes. Note that there are two ways of predicting T: one in which C is the signature gene matrix corresponding to a selected fraction of marker genes and varies when the fraction changes, and another in which C is the signature gene matrix corresponding to the whole set of marker genes and does not change with different selected fractions. The PCC_T_ corresponding to these two situations was named PCC_T1_ and PCC_T2_, respectively. In general, PCC_p_ increased with the inclusion of more marker genes, and the increase was relatively sharp before the inclusion of the top 25% of marker genes. Interestingly, before the inclusion of the top 25% of marker genes, PCC_T1_ and PCC_P_ were negatively correlated, whereas PCC_T2_ and PCC_P_ were positively correlated (Figure 1C). Although for a given method, a higher PCC_T2_ usually indicates a higher PCC_P_, this prediction cannot be generalized when the comparison is across different methods (Figure 1D). Accordingly, we concluded that it is not possible to select a deconvolution method by comparing their PCC_T_.

In our situation of deconvolution, the reference expression profiles were obtained from a single-cell study of the placenta (Vento-Tormo et al., 2018) while the bulk data were from a large cohort of microarray study on PE (Leavey et al., 2016). In order to select a deconvolution method from the recommended ones, we, therefore, prepared three benchmark tests whose degree of deconvolution difficulty was considered to be similar to ours and reasoned that a method performing stably across these three datasets would also likely perform well in our situation. In the first benchmark dataset (Test 1), the bulk data were PBMC RNA-seq data produced by Finotello et al. (2019), and the reference expression profiles were from the single-cell PBMC RNA-seq data generated by Ding et al. (2020) using 10X sequencing platform. In the second benchmark dataset (Test 2), the reference expression profiles were the same as in Test 1, while the bulk data were PBMC microarray data (Newman et al., 2015). In the third benchmark dataset (Test 3), the bulk data were the same as in Test 2, while the reference expression profiles were from the single-cell PBMC RNA-seq data generated by Ding et al. (2020) using Drop-seq and inDrops sequencing platform. In each of the three benchmarks, the signature gene matrices were produced from a top fraction of marker genes selected according to the previously described procedures. In general, most bulk reference-based methods perform better when more marker genes are used, and CIBERSORT and RLR achieved better performance than the other three methods did across the three tests (Figure 1E). To further quantify how stable a method’s performance is with the inclusion of more marker genes, we ranked the performance of the five methods at a given fraction (from top 25% to top 100%) of marker genes and then calculated the averaged rank of each method. We found that CIBERSORT had the most stable overall performance across the three tests (Figure 1F). We also evaluated the performance of four single-cell reference-based methods (DWLS, MuSiC, SCDC, and CIBERSORTx) in these three tests and found that DWLS performed the best among the four methods though its overall performance was worse than CIBERSORT’s (Figure 1G).

Based on the above analyses, we, therefore, developed a practical pipeline for the deconvolution of PE microarray data. We would follow the procedures described previously to select marker genes and construct a signature gene matrix. Then, we would use CIBERSORT, the method with the most stable and good performance across the three benchmark tests, to perform deconvolution.

### 2.2 Deconvolution of Preeclampsia Placenta Microarray Data Revealed Significantly Altered Proportions of Trophoblasts in Preeclampsia

**FIGURE 2:**
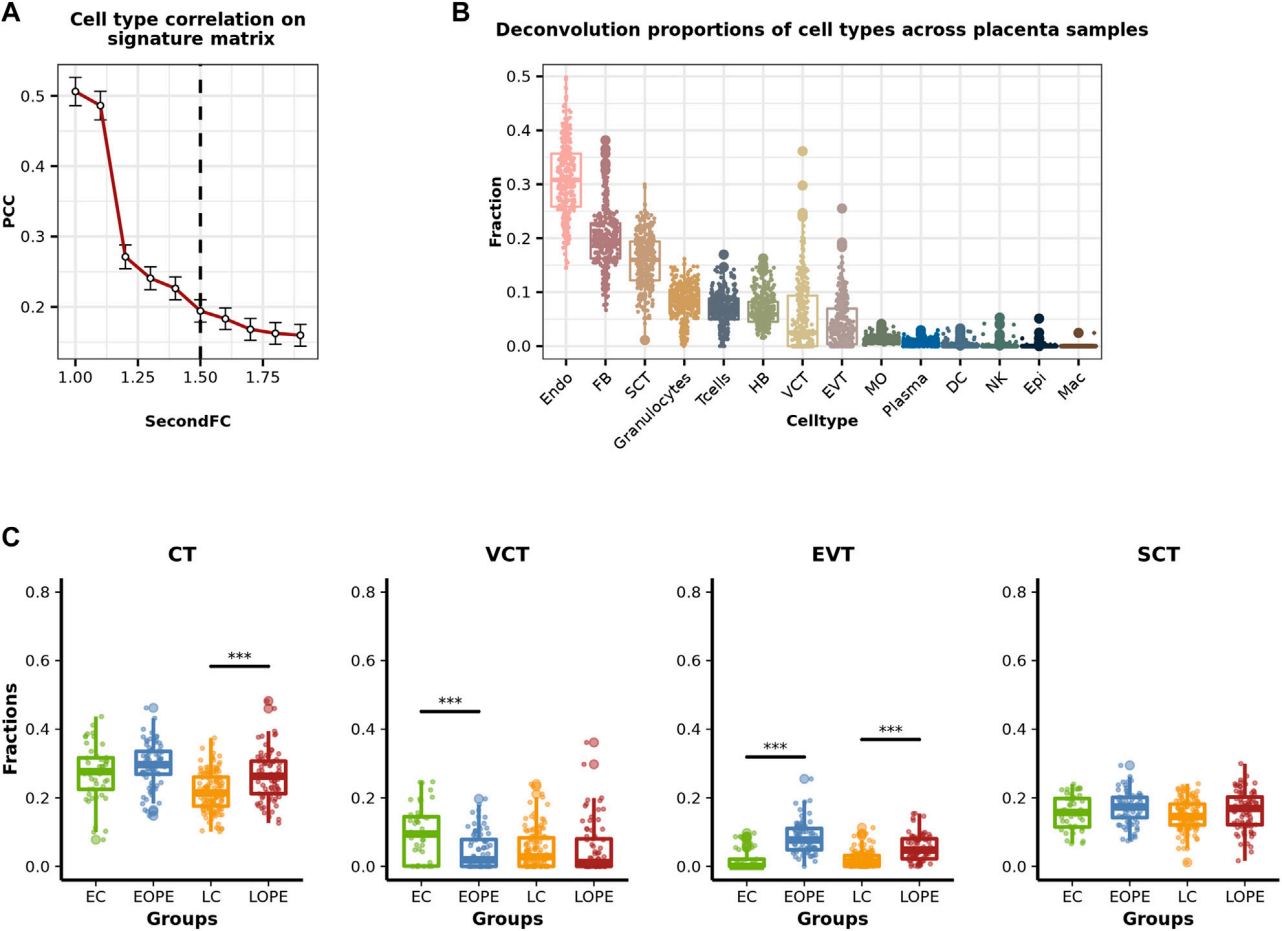
Estimated cell-type proportions of placental samples included in the cohort of placental microarray data. **(A)** Average PCC on placenta signature matrix changing with SecondFC cutoff. **(B)** Boxplots of the estimated proportions of different placental cell types in the cohort of placenta microarray data. **(C)** Comparison of the estimated proportions of trophoblasts between PE and normal samples. CT: cytotrophoblast, EC: early-stage control, EOPE: early-onset preeclampsia, LC: late-stage control, LOPE: late-onset preeclampsia.

The cohort of PE placental microarray data was constructed by Leavey et al. (2016) and included a total number of 330 samples (157 PE and 173 control), of which 157 had detailed clinical information. The clinical information is mainly about the fetal and maternal state, like newborn weight z-score, maximum systolic bp, mode proteinuria, etc. The reference expression profiles were obtained from the single-cell placental RNA-seq data produced by Vento-Tormo et al. (2018). Following Francisco’s suggestion to include all cell types that possibly exist in the bulk mixture, we selected the expression profiles of all major cell types (subpopulations were pooled) in the placenta and the blood of the Vento-Tormo dataset (see Section 3 for details) and constructed a signature matrix consisting of endothelial cells (Endo), epithelial cells (Epi), fibroblasts (FB), three types of trophoblasts cells—villous cytotrophoblasts (VCT), syncytiotrophoblasts (SCT), and extravillous trophoblasts (EVT), and eight types of immune cells—Hofbauer (HB), natural killer (NK), T cells, plasma, granulocytes, monocyte (MO), macrophage (Mac), and dendritic cells (DC). Here, we set SecondFC to 1.5 (Figure 2A) by following the above-described procedures to select the marker genes for deconvolution and applied CIBERSORT to perform the deconvolution. The deconvolution results showed that Endo, the major component cells of placental blood vessels, were the largest population of cells in the placenta samples of this cohort, while FB, which is located within the villus core matrix with HB, and SCT were the second and the third largest population of cells, respectively (Figure 2B). However, if VCT and EVT were considered together with SCT, then trophoblasts were the largest population of cells in the placenta. Among the eight types of immune cells, however, only granulocytes and T cells accounted for a noticeable proportion in the placental samples (Figure 2B).

PE can be generally classified as early-onset PE (EOPE) and late-onset PE (LOPE) depending on the gestational age (GA) (34 weeks) of disease onset (Von Dadelszen et al., 2003). Following this definition, we then classified the PE samples in this cohort as EOPE or LOPE and also classified the normal samples as early control (EC) or late control (LC), respectively. As trophoblasts are the major population of cells in the placenta and are also responsible for the normal function of the placenta, we compared the proportion of trophoblasts between PE and normal controls and observed significant differences (Figure 2C). LOPE has a significantly higher proportion of trophoblasts than its group of normal controls (Figure 2C). As for the subpopulations of trophoblasts, compared to normal controls, VCT’s proportion was significantly lower in EOPE and lower but not significant in LOPE; EVT’s proportion was significantly higher in both EOPE and LOPE; SCT’s proportion was not significantly altered in PE (Figure 2C). It has been shown that the impaired invasive ability of EVT is a major reason for dysfunctional placenta in PE (Crosley et al., 2013). Here, the significantly increased proportion of EVT in PE may be because of a compensatory enhancement of EVT production occurring in response to dysfunctional EVT.

### 2.3 The Predicted Cell-Type-Level Expression Profiles Revealed Patterns of Cell-Type-Specific Gene Expression Alterations in Preeclampsia

**FIGURE 3:**
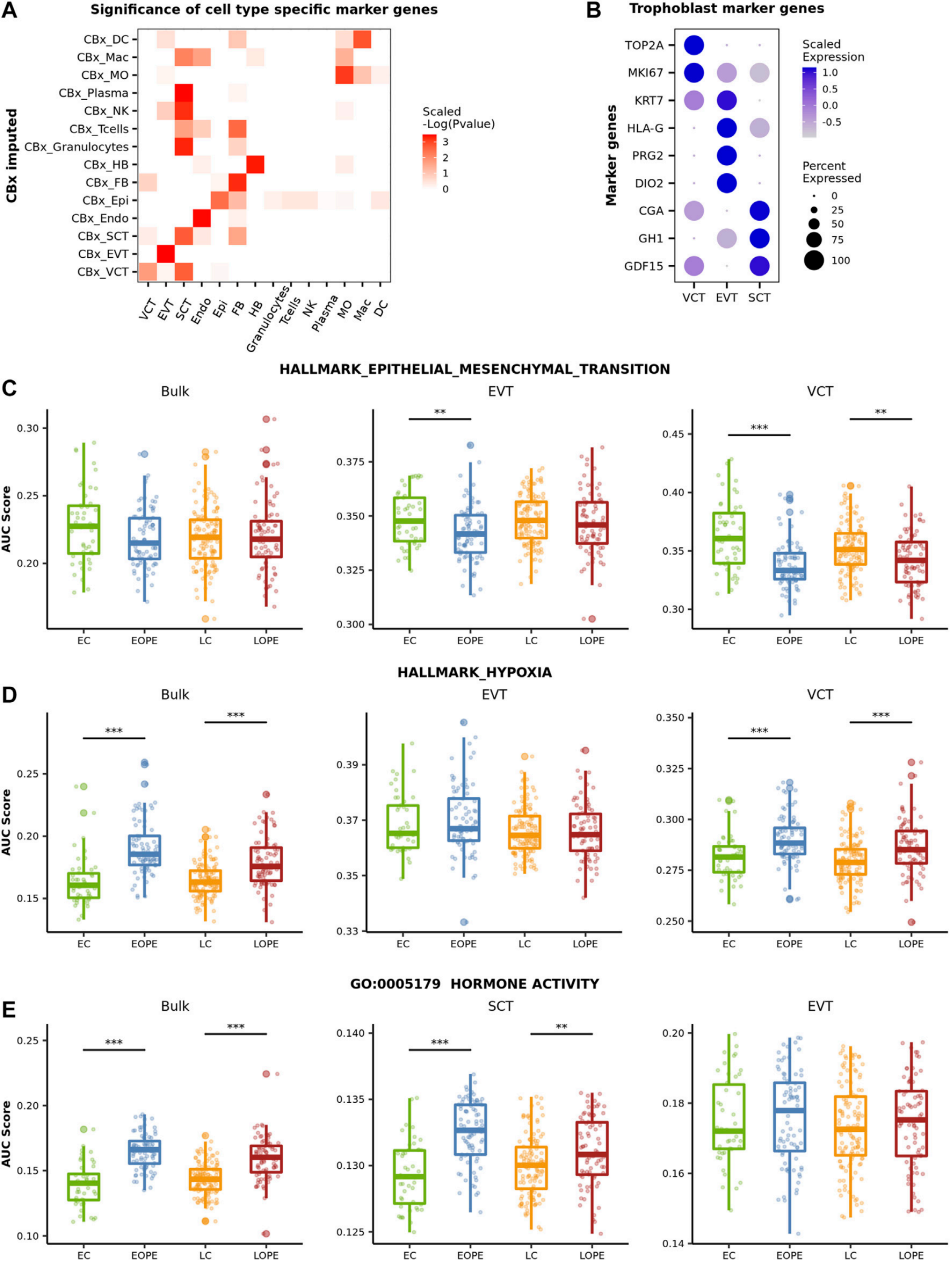
Comparison of the activity of canonical PE-related pathways in between PE and normal samples. **(A)** Assessment of the biological relevance of the predicted cell-type-level expression profiles. We first averaged the expression profile of imputed transcriptome in each cell type. Then, the Wilcox test was used to evaluate if the expression on the averaged profile of the cell-type marker genes is specifically high in the corresponding cell type. The negative log *P* value of the Wilcox test was scaled by rows. **(B)** The expression level of classic trophoblasts marker genes in the predicted cell-type-level expression profiles of three trophoblast subtypes. **(C–E)** Activity of the canonical PE-related pathway in different groups of samples. The activity was measured by AUCell.

Given the estimated cell-type proportions, CIBERSORTx provides a way to infer cell-type-level expression profiles (Steen et al., 2020). Here, we applied the high-resolution mode of CIBERSORTx with the default parameters to predict the expression profiles of placental cell types for each sample. To validate that the predicted cell-type-level expression profiles are biologically meaningful, we tested whether the corresponding cell-type-specific marker genes identified from the reference expression profiles were at significantly higher expression levels than background genes did. The biological relevance of the predicted expression profiles of trophoblasts (VCT, EVT, and SCT), Endo, Epi, FB, and HB was well validated (Figure 3A). However, the predicted expression profiles of granulocytes, T cells, NK, and plasma were found to be more similar to SCT’s than to themselves (Figure 3A), indicating that the predicted expression profiles of these cells are likely not very useful for further analysis. We further examined the expression levels of the canonical marker genes of the three trophoblast subtypes in these profiles. As the trophoblast stem cell, VCT highly expresses *TOP2A* and *MIK67*, both of which are related to cell proliferation, and the keratin gene *KRT7* is highly expressed in EVT too. The other marker genes of EVT are *HLA-G*, which is involved in the immune tolerance process (Ferreira et al., 2017), and *PRG2* and *DIO2*, both of which are related to the invasion ability of EVT (Windsperger et al., 2017; Adu-Gyamfi et al., 2021). SCT highly expresses *CGA* and *GH1*, which are related to hormone synthesis (Freemark, 2010), and *GDF15*, a classic SCT marker gene, was reported to be associated with PE (Sugulle et al., 2009). Here, these selected marker genes were all highly expressed in their respective predicted cell-type-specific expression profiles (Figure 3B). As such, the aforementioned results indicated that the predicted expression profiles of major placental cell types, including Endo, FB, HB, and trophoblasts were worthy of further exploration.

We then focused on the predicted expression profiles of trophoblasts and inspected the activity of several canonical PE-related pathways in between PE and normal controls. As a comparison, we also inspected the activity of these pathways by using the bulk expression profiles. Here, the activity of a pathway was measured by AUCell (Aibar et al., 2017). AUCell sorts all genes in the sample according to their expression and calculates the pathway activity of each sample according to the ranks of the pathway genes. The canonical PE-related pathways inspected here include the epithelial-mesenchymal transition (EMT) hallmark pathway, the hypoxic pathway, and the GO pathway of “Hormone activity.”

During the development of trophoblasts (from VCT to EVT and from noninvasive EVT to invasive EVT), the cell undergoes phenotypic changes termed the EMT process in order to gain the invasive ability (Vićovac and Aplin, 1996). It has been well established that the EMT process of trophoblasts was inhibited in PE (Sun et al., 2011). Using the bulk data, however, we did not observe any significant difference in EMT’s activity between PE and normal samples (Figure 3C). In contrast, in both EVT and VCT, the activity of the EMT pathway was significantly reduced in both EOPE and LOPE though the reduction was not significant in LOPE’s EVT (Figure 3C), indicating that the invasive ability of EVT and the differentiation of VCT to EVT are likely both inhibited in PE. Not that no EMT-related genes were predicted in SCT.

Placenta hypoxia is one of the most significant clinical manifestations of PE (Soleymanlou et al., 2005). This was clearly shown by using the bulk data: the activity of the hypoxia pathway was significantly upregulated in PE samples (Figure 3D). The predicted cell-type-level expression profiles provided more detailed information about hypoxia at the cellular level. In both EOPE and LOPE, the activity of the hypoxia pathway was significantly upregulated in VCT, but not in EVT (Figure 3D), reflecting the different pressure of oxygen limitation to different types of trophoblast cells. The significant upregulation of the hypoxia pathway in VCT is probably because VCT is located deeply in the trophoblast layer and is more likely affected by oxygen limitation. Note that there were only a few genes predicted to be associated with the hypoxia pathway in SCT.

It has been reported that the placenta of PE is likely hormonally compensated in response to development deficiency (Tamimi et al., 2003). Here, we observed a significantly higher “Hormone activity” in PE by using the bulk data and further found that the activity was significantly upregulated in SCT, but not in EVT and VCT, by using the predicted cell-type-level expression profiles (Figure 3E). Thus, the above results showed that the predicted cell-type-level expression profiles revealed patterns of cell-type-specific gene expression alterations in PE.

**FIGURE 4:**
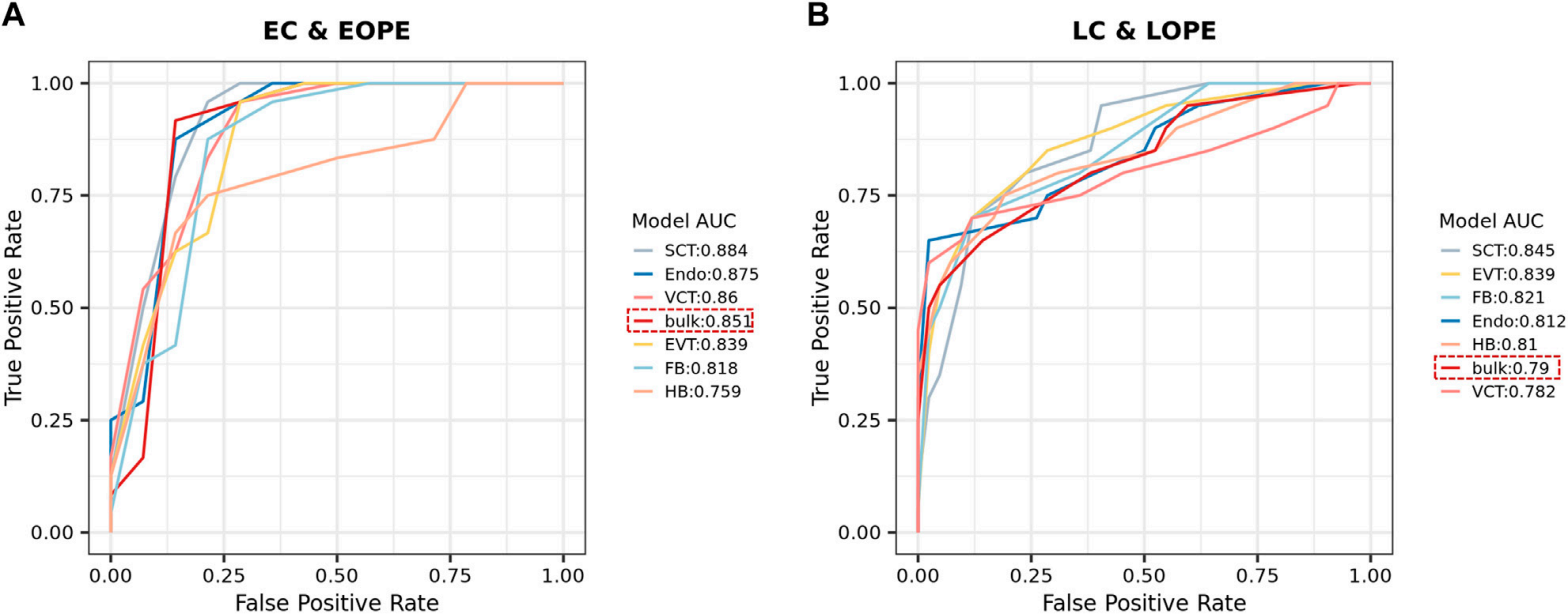
**(A,B)** ROC of SVM models for distinguishing EOPE **(A)** and LOPE **(B)** from their respective groups of normal samples. SVM models were trained by using either the bulk or the predicted cell-type-level expression profiles.

As the predicted cell-type-level expression profiles were biologically relevant and provided more details about the altered PE canonical pathways, we explored whether they could better distinguish PE from normal controls than the bulk expression profiles did. For each of the six above-mentioned cell types, we used 80% samples to train an SVM model to distinguish PE from normal samples by using the predicted cell-type-level expression profiles and then tested it using the 20% remaining samples (see Section 3 for details about the procedures). As a comparison, we also used the bulk expression profiles to develop an SVM model. Overall, it was easier to distinguish EOPE from LOPE; for most cell-type-level SVMs, their performance was comparable to that of bulk-level SVM in EOPE but was superior to LOPE (Figure 4A,B). However, even the best SVM in either EOPE or LOPE only achieved an AUC_ROC_ less than 0.9, indicating that PE is a heterogeneous and complex disease that may involve multiple subtypes and cannot be easily described by using one model.

### 2.4 Unsupervised Clustering of Predicted Cell-Type-Level Expression Profiles Revealed Clinically Distinct Preeclampsia Subgroups

Although EOPE is generally considered more severe than LOPE, the real situation is usually more complex and severe and nonsevere PE types are actually difficult to distinguish by their subjective clinical indicators (Roberts et al., 2021). The cohort of PE microarray data provided 13 clinical features for a total number of 157 PE samples (EOPE: 80 and LOPE: 77). These features can be divided into two general categories: fetal state-related and maternal state-related. The fetal state-related features include GA, newborn weight z-score, placental weight z-score, umbilical cord diameter, mean umbilical PI, Apgar score (1 min), Apgar score (5 min), and IUGR diagnosis, while the maternal state-related features include maximum systolic bp, maximum diastolic bp, mode proteinuria, mean uterine pi, and maternal BMI. To explore whether PE samples could be classified into subtypes, here for each of the six placental cell types, we conducted unsupervised clustering of PE samples using their predicted expression profiles. Then, we investigated whether the clustering was significantly associated with each of the 13 clinical features.

**FIGURE 5:**
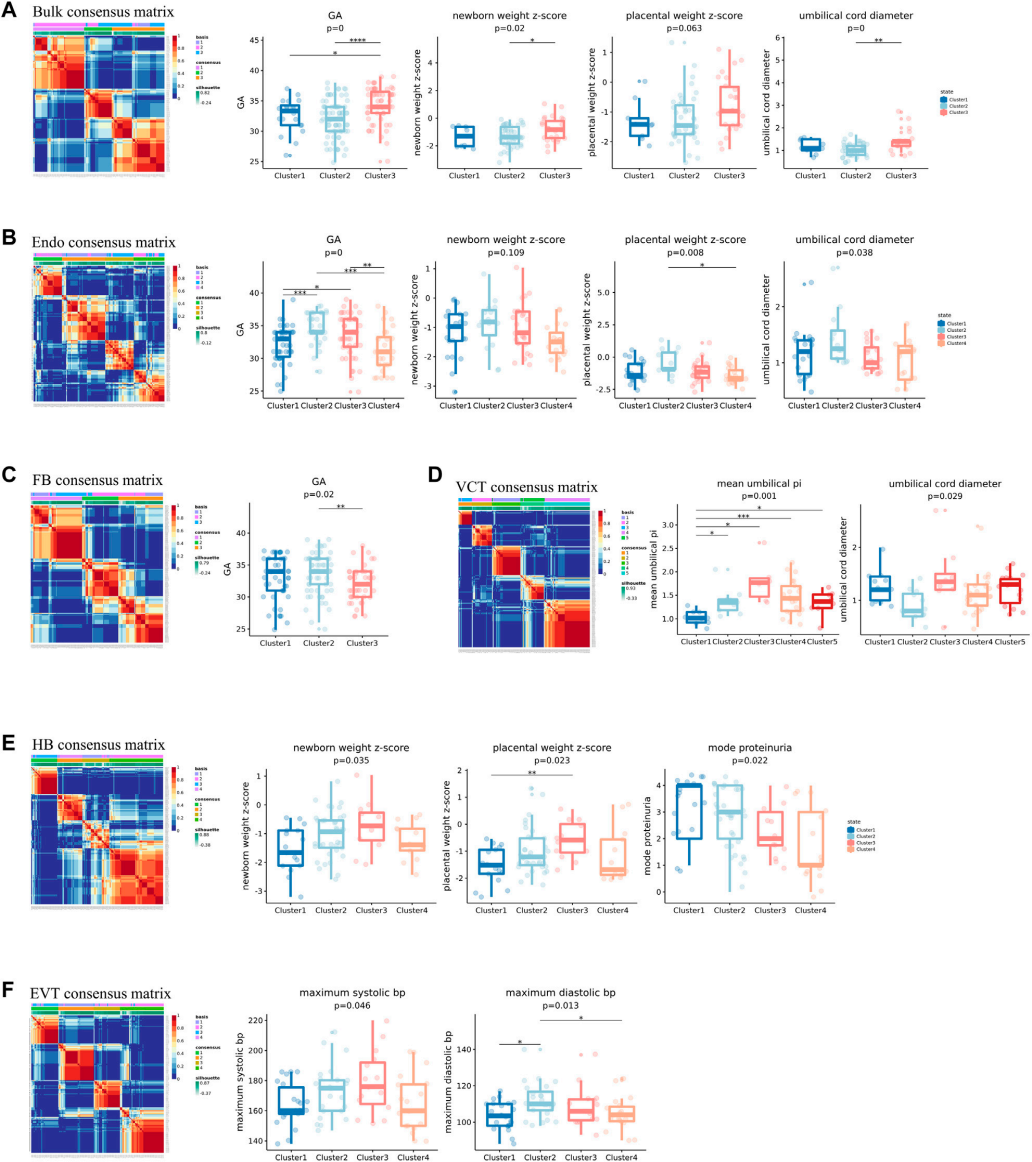
Unsupervised clustering of PE samples using the bulk or predicted cell-type-level expression profiles. The expression profiles used in **(A–F)** were the bulk, the predicted cell-type-level expression profiles of Endo, FB, VCT, HB and EVT, respectively. In each of **(A–F)**, the clinical phenotypes significantly associated with the clustering were shown.

As a comparison, we first conducted unsupervised clustering of PE samples based on their bulk expression profiles by using negative matrix factorization (NMF) (see Section 3 for details). We obtained three clusters. The clustering results were found to be significantly associated with not only the definition of EOPE and LOPE but also four fetal state-related features: GA, newborn weight, placental weight, and umbilical cord diameter (Figure 5A). Because EOPE and LOPE are defined based on their GA while newborn weight, placental weight, and umbilical cord diameter are also strongly dependent on GA, it is not unexpected that those features were all significantly associated with the clustering results. However, we did not observe any significant maternal state-related clinical features associated with the clustering results.

We next conducted unsupervised clustering of PE samples using the predicted cell-type-level expression profiles of each of the six cell types and investigated their association with clinical features. We found that the clustering results of all six cell types except for SCT were all significantly associated with some clinical features (Figure 5). The reason why SCT was not linked to any clinical features was probably that some transcriptional signatures of SCT were misassigned to other cell types, such as NK, granulocytes, and plasma. The clinical features linked to Endo, FB, and VCT were all fetal state-related: Endo was linked to GA, newborn weight z-score, placental weight z-score, and umbilical cord diameter; FB was linked to GA; VCT was linked to mean umbilical PI and umbilical cord diameter (Figures 5B–D). Interestingly, the clinical features linked to HB were both fetal state and maternal state-related: newborn weight z-score, placental weight z-score, mode proteinuria, and IUGR diagnosis, while the clinical features linked to EVT were only maternal-related: maximum systolic bp, and maximum diastolic bp (Figures 5E,F). HB is an immune cell that promotes trophoblast differentiation and angiogenesis by producing various growth factors and cytokines (Wang and Zhao, 2010). EVT is the primary cell type in the placenta that invades the decidual of the mother during the pregnancy. The reasons why these 2 cell types were linked to maternal state-related features were probably because they had more interaction with maternal cells. In contrast, Endo, FB, and VCT may be more related to the growth of the placenta, that is, more fetus oriented. The predicted cell-type-level expression profiles thus provided more links to clinical features that would not be observed by using the bulk expression profiles, especially the maternal state-related features.

**FIGURE 6:**
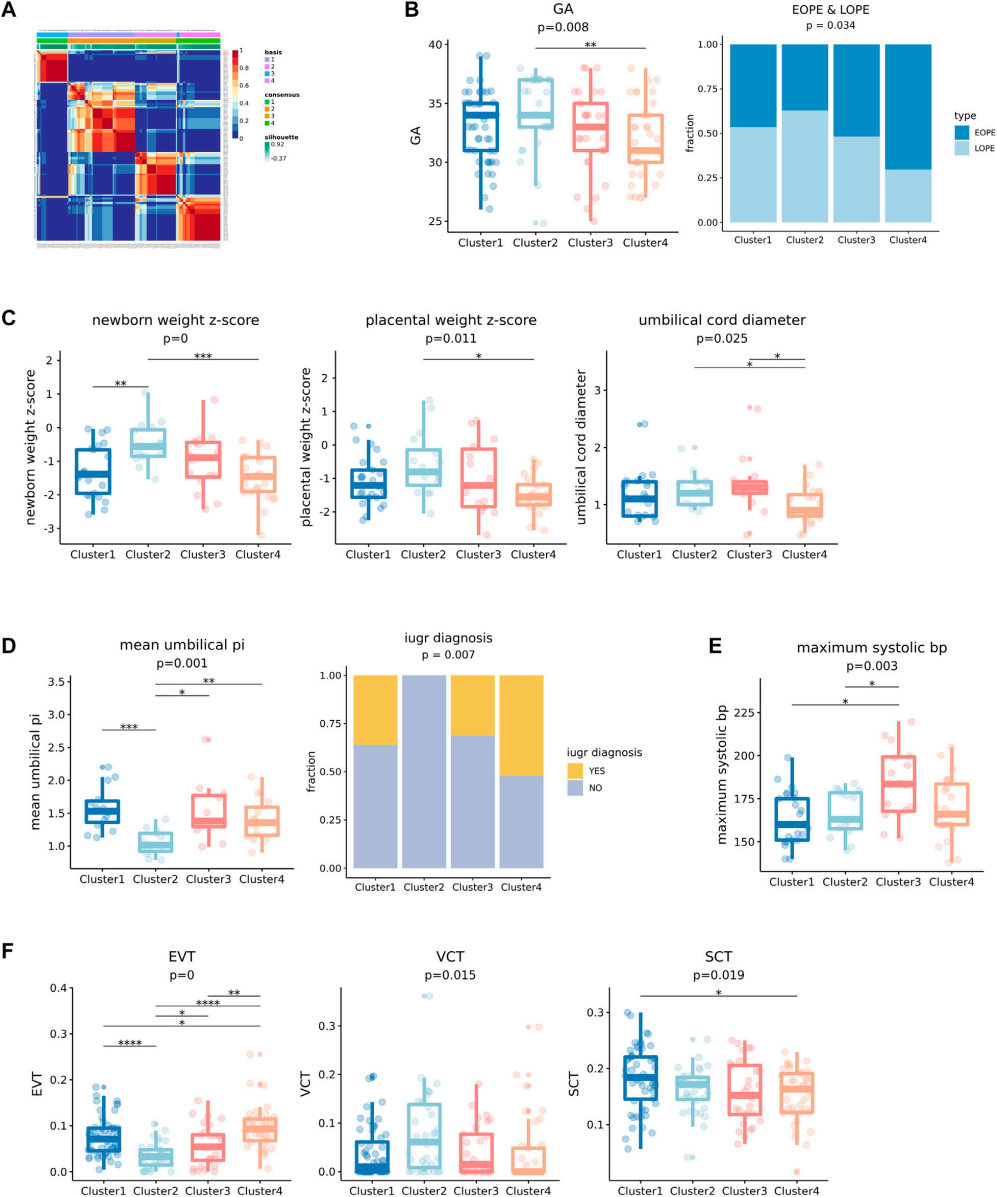
Unsupervised clustering results of integrated five cell types of transcriptional profiles of PE by NMF and significance testing of clinical features. **(A)** Unsupervised clustering result of integrated cell types transcription profiles using NMF, the consensus matrix of NMF output display that four stable clusters can be obtained. **(B)** The four clusters showed significant differences in gestational age (GA), and the fraction of EOPE and LOPE. **(C)** The four clusters showed significant differences in newborn weight z-score, placental weight z-score, and umbilical cord diameter which reflect the state of fetal development. **(D)** The four clusters showed significant differences in mean umbilical PI and the fraction of IUGR in the cluster. A higher mean umbilical PI indicates a greater likelihood of IUGR. **(E)** Four clusters showed significant differences in maximum systolic bp. **(F)** Differences in the proportion of three kinds of trophoblast cells (EVT, VCT, and SCT) in four clusters of PE.

Given that the predicted cell-type-level expression profiles of the above five cell types were strongly linked to clinical features, we constructed an integrated expression profile for each sample by combining the predicted expression profiles of the highly variable genes of each cell type and then conducted unsupervised clustering (see Section 3 for details about constructing the integrated expression profiles). We obtained four clusters by using NMF (Figure 6A) and found that they were significantly associated with seven clinical features of which six were fetal state-related (GA, newborn weight z-score, placental weight z-score, umbilical cord diameter, mean umbilical PI, and IUGR diagnosis) and one was maternal state-related (maximum systolic bp) (Figures 6B–E). We compared each of these significant features between the four PE clusters and found that they had distinct clinical features. In general, Clusters 1 and 2 have longer GA, while Clusters 3 and 4 have shorter GA, with Clusters 2 and 4 having the longest and the shortest GA, respectively (Figure 6B). Clusters 2 and 4 are also significantly enriched with LOPE and EOPE samples, respectively, while the other two clusters do not have a preference for either EOPE or LOPE (Figure 6B). Probably because Clusters 2 and 4 have the longest and shortest GA, they also correspond to the best and the poorest fetal state, respectively (Figure 6C). Although Cluster 1’s GA is close to Cluster 2’s, its fetal state was significantly worse than that of Cluster 2.

For example, Cluster 1 has a significantly higher proportion of intrauterine growth retardation (IUGR), which consists of the higher “mean umbilical PI”—a potential IUGR predictor (Khanduri et al., 2017), compared to Cluster 2 (Figure 6D). And its other fetal-related features are also significantly worse than Cluster 2’s (Figure 6C). Cluster 3’s GA is close to Cluster 4’s, but it is significantly maternal state-related: it has the highest maximum systolic bp, that is, the most severe state of blood pressure (Figure 6E). We also found that the proportions of EVT and VCT were significantly different in these four clusters. For example, the proportion of EVT was the lowest in Cluster 2 which corresponds to the best fetal state, while the proportion of VCT was the highest (Figure 6F). Note that when comparing PE samples with normal controls, we observed a significantly increased proportion of EVT and decreased proportion of VCT in PE samples. Therefore, the relative increase or decrease of the proportion of EVT may indicate the severity of PE.

**FIGURE 7:**
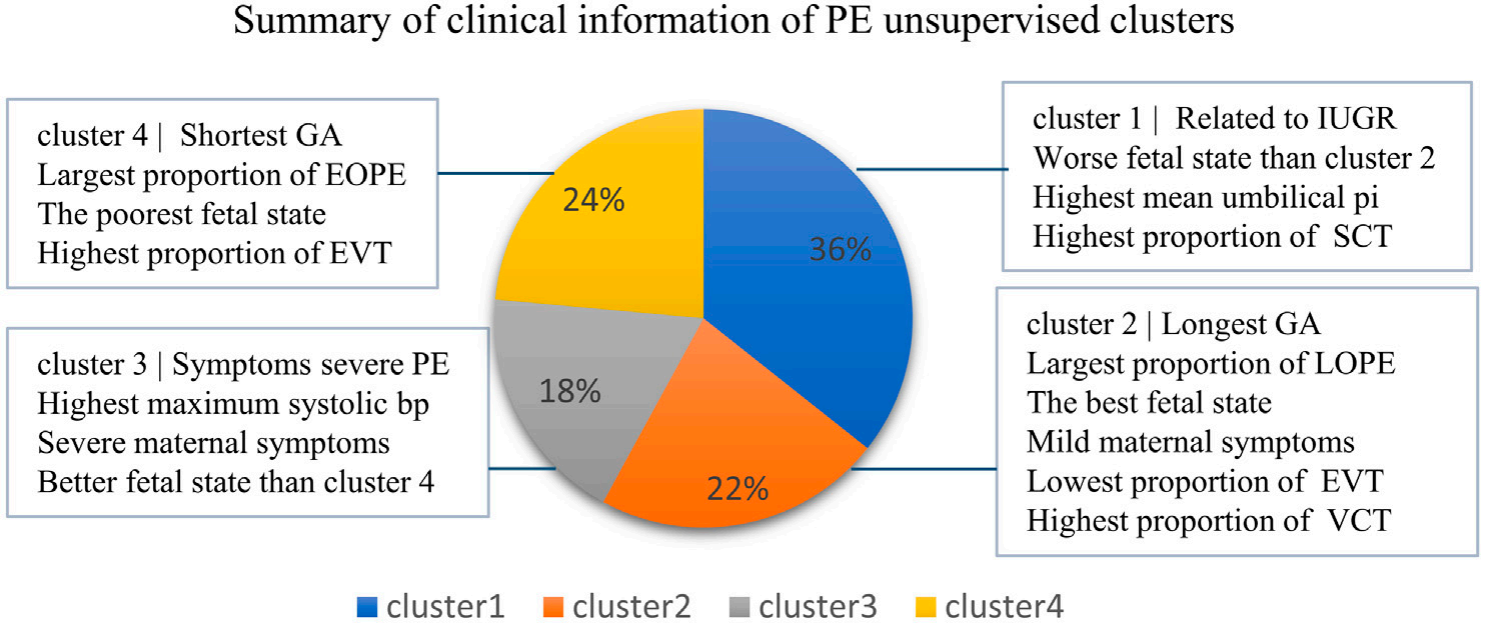
Information overview of four clusters of PE. The sample fractions of the four clusters, respectively, accounted for 36%, 22%, 18%, and 24%.

In conclusion, by using the integrated expression profiles, we obtained four clinically distinct PE subtypes that are significantly associated with not only fetal state-related but also maternal state-related clinical features that would not be observed by using the bulk expression profiles (Figure 7), highlighting the important value of deconvolution.

## 3 Materials and Methods

### 3.1 Datasets Used in This Study

A number of PBMC datasets were used for developing the deconvolution pipeline. The bulk PBMC datasets included Finotello’s PBMC RNA-seq dataset (Finotello et al., 2019) (GSE107572) and Newman’s PBMC microarray dataset (Newman et al., 2015) (GSE65136), and both datasets had known flow-sorting cell-type proportions. The datasets for the reference expression profiles included Hoek’s PBMC data (Hoek et al., 2015) with cell-type purified RNA-seq data (GSE64655) and Ding’s PBMC dataset (Ding et al., 2020) (https://singlecell.broadinstitute.org/single_cell/study/SCP424) that includes single-cell data produced by 10X, Drop-seq, and inDrops sequencing platforms. The cohort of placenta microarray dataset was built by Leavey et al. (2016) (GSE75010), integrating from 8 placenta microarray studies. It contains 157 samples that had detailed clinical information, including fetal state-related and maternal state-related indicators, and the single-cell placenta reference was generated by Vento-Tormo et al. (2018) (https://www.ebi.ac.uk/arrayexpress/experiments, E-MTAB-6678, E-MTAB-6701). Datasets from GEO were downloaded with accessions above through the website (https://www.ncbi.nlm.nih.gov/geo).

### 3.2 Procedures for Constructing the Signature Gene Matrix and Description of the Deconvolution Methods Used in the Evaluation

We followed Francisco’s recommended strategy on marker gene selection. Given a single-cell reference gene expression matrix, we applied the following parameters to select the marker gene set: logFC ≥ 1 and logCPM ≥ 1. For SecondFC, we determined the relationship between SecondFC and the average correlation between cell types in the signature gene matrix, setting it to no less than 6. Here, logFC means log fold change between the highest expressed cell type and the average expression of other cell types, logCPM means the log average normalized expression level among all cell types, and SecondFC means the average expression fold change of a given marker gene between the highest expressed cell type and the second-highest expressed cell type. When evaluating different deconvolution methods, we ranked the marker genes by logFC and selected a given fraction of top-ranked genes, for example, top 5%, 10%, … 100%, and averaged the expression counts of all cells in each cell type to construct the signature gene matrix for the selected marker genes.

We evaluated nine deconvolution methods in this study, among which five bulk reference-based methods and three single-cell reference-based methods were recommended by Francisco et al. (Cobos et al., 2020). The five bulk reference-based methods are nonnegative least squares (NNLS) (https://CRAN.R-project.org/package=nnls), ordinary least squares (OLS) (https://www.R-project.org/), robust linear regression (RLR) (https://www.stats.ox.ac.uk/pub/MASS4/), FARDEEP (https://github.com/YuningHao/FARDEEP), and CIBERSORT (https://cibersort.stanford.edu/), while the three single-cell reference-based methods are DWLS (https://github.com/dtsoucas/DWLS), MuSiC (https://github.com/xuranw/MuSiC), and SCDC (https://github.com/meichendong/SCDC). In addition, we added CIBERSORTx (https://cibersortx.stanford.edu/), which is based on CIBERSORT’s improved method of using single-cell data as input.

### 3.3 The Processing of the Single-Cell Placental Atlas

The single-cell reference expression matrix used for the deconvolution of placental microarray data was constructed from the single-cell placental atlas produced by Vento-Tormo et al. (2018). In order to reduce the problem of collinearity, that is, challenging to the deconvolution algorithm, we merged the subgroups of each of the following cell types in the Vento-Tormo dataset: “DC1” and “DC2” were merged into DC (dendritic) cells, “dNK p,” “dNK1,” “dNK2,” “dNK3,” “NK CD16-,” and “NK CD16+” were merged into NK (natural killer), “dM1,” “dM2,” and “dM3” were combined to Mac (macrophage), “Endo (f),” “Endo (m),” and “Endo L” were merged into Endo (endothelial), “Epi1” and “Epi2” were merged into Epi (epithelial), and “fFB1” and “fFB2” were merged into FB (fibroblast). Finally, the single-cell reference expression matrix consisting of a total number of 14 placental cell types was constructed, including eight types of immune cells Hofbauer (HB), NK, T cells, plasma, granulocytes, monocyte (MO), Mac, and DC), three subtypes of trophoblasts (VCT, EVT, and SCT), Epi, Endo, and FB cells. The signature gene matrix was then constructed by applying these cutoffs (logFC ≥ 1, logCPM ≥ 1, and SecondFC ≥ 1.5) and by requiring that each marker gene was expressed in at least 30% of cells of the corresponding cell type.

### 3.4 The Development of SVM Models to Distinguish PE From Normal Controls

We randomly selected 80% of the samples (training set) to train an SVM model and tested the model using the 20% remaining samples. When training the SVM model, we first identified the differentially expressed genes (DEGs) between PE and normal controls by controlling log CPM >4 using the package of “edgeR” in R. The log-normalized expression profiles of DEGs were then used as the input to train SVM model. For the SVM model, we used svm.SVC classifiers from the scikit-learn library in Python. For the kernel, we chose “linear”. For other parameters like degree and gamma, we used the default parameters in the function svm.SVC. For the hyperparameter, C was grid searching between 0 and 2, with 0.2 intervals, and fivefold cross-validation was performed on the training set to find the most appropriate hyperparameter C. The hyperparameter C was determined and then retrained for the whole training set and tested on the test set.

### 3.5 Procedures of Unsupervised Clustering of Bulk or Predicted Cell-Type-Level Expression Profiles

We first log-normalized raw expression counts and selected highly variable genes by using the “mean.var.plot” method in the Seurat package, with the parameter “mean.cutoff” > 0.5. The “dispersion.cutoff” parameter was tried between 1 and 2.5, with 0.1 intervals, to ensure the stability of unsupervised clustering results. Next, we used the “ScaleData” method to scale the data to maximize the variation between samples. Finally, we used the negative matrix factorization (NMF) to do unsupervised clustering. The input of NMF was the scaled data, and the output of the NMF was the specified k clusters, where k is given artificially. To determine the optimal number of clusters, we iteratively tested k from 2 to 10. In each iteration, we calculated the cophenetic coefficient (CC) of the clusters, which represents the stability of clustering. Ideally, CC remains stable initially when k increases from 2 and then drops quickly when k continues to increase, and the k before the quick drop of CC would be selected. In practice, we would try different values for “dispersion.cutoff” when selecting highly variable genes and selected the one where we could identify the best k.

### 3.6 Statistical Tests to Inspect the Association of Clinical Features with the Clustering of Preeclampsia Samples

Most of the clinical features are in numerical values. To test the significance of the association of a clinical feature with the clustering of PE samples, when the data type of the clinical feature is numerical, we used analysis of variance (ANOVA) to inspect whether there is any difference in the mean of the clinical features in between clusters, and used *t*-test to check the difference between pairs of clusters, where *p* values were corrected by FDR; when the data type is categorical, we used the Chi-Square test.

## 4 Discussion

In this study, we aimed to reanalyze a large cohort of PE placental microarray data through deconvolution. For this purpose, we first attempted to develop a practical pipeline by experimenting with the strategies for marker gene selection and several deconvolution methods recommended by Francisco’s benchmark study (Cobos et al., 2020). While the selection of marker genes was relatively straightforward, we found that it was not possible to determine which deconvolution method to use by using the metric of PCC_T_, the PCC between the predicted expression and true expression of bulk transcripts that can be calculated given estimated cell-type proportions. To have an approximate solution to this problem, we designed several benchmark tests that likely have a similar degree of challenges to the deconvolution of PE placental microarray data and found CIBERSORT performed the best across these tests. CIBERSORT was therefore chosen as the deconvolution method of our study. The successful validation of the biological relevance of the predicted cell-type-level expression profiles of the major placental cell types using their marker genes also confirmed that the deconvolution results by CIBERSORT can be trusted. Based on our experience, the CIBERSORT-based practical pipeline may also well be suited for the deconvolution of other microarray datasets.

In this study, the deconvolution of PE placental microarray data has resulted in several important findings of PE. First, the proportions of EVT and VCT in the placenta are significantly altered in PE, but in different directions, with EVT increasing and VCT decreasing. It has been shown that the differentiation of VCT to EVT and the transition of EVT to gain invasive ability are both inhibited by PE (Sun et al., 2011). Consistently, the activity of the EMT pathway, which plays an important role in these two important development processes (Vićovac and Aplin, 1996), was found to be significantly downregulated in both VCT and EVT in this study. Therefore, the significant increase in EVT and the significant decrease of VCT likely reflect a compensatory enhancement of EVT differentiation and transition in response to the impaired invasive abilities of EVTs. Second, the canonical PE-related pathways showed cell-type-specific alterations in PE. For example, hypoxia was mainly found in VCT, while enhanced hormonal production was found in SCT. Third, placental cell types could be linked to not only fetal state but also maternal state-related clinical features by clustering of predicted cell-type-level expression profiles. In contrast, the clustering of bulk expression profiles could be only linked to fetal state-related clinical features. Although the placenta is a fetus tissue, PE is a disease with significant maternal symptoms, such as high blood pressure. It is therefore of great value that placental cell types, specifically EVT, could be linked to maternal state-related features in our study. Fourth, four clinically distinct clusters of PE samples were identified in this study and likely represent distinct PE subtypes. Clusters 2 and 4 have the longest and the shortest GA and also correspond to the best and the poorest fetal state, respectively. Although Cluster 1 has a similar GA to Cluster 2, it has a significantly much worse fetal state. As for Cluster 3, though it has a similar GA to Cluster 4, it has the most severe maternal state, with the highest blood pressure among the four clusters.

The discovery of clinically distinct clusters by this study is of great value to the field of PE. For example, a new diagnostic model can be developed based on the classification of these clinically distinct clusters, such that PE patients can be assigned into different groups and different treatment plans can be applied. New therapeutic drugs targeting the most severe PE may also be developed by selecting drug target genes from the marker genes from the PE cluster with the most severe outcomes. Moreover, there is a rich trove of bulk RNA-seq or microarray data in the public domain, with many having disease-related clinical information. The fact that the deconvolution of PE placental microarray data led to several new findings on the disease strongly suggests that similar deconvolution studies should be conducted to reanalyze disease-related bulk data to generate new insights into the molecular mechanisms of diseases.

## Data Availability

Publicly available datasets were analyzed in this study. This data can be found here: https://www.ncbi.nlm.nih.gov/geo/query/acc.cgi?acc=GSE75010.
